# Psychological Resources Protect Well-Being During the COVID-19 Pandemic: A Longitudinal Study During the French Lockdown

**DOI:** 10.3389/fpsyg.2020.590276

**Published:** 2020-12-04

**Authors:** Nicolas Pellerin, Eric Raufaste

**Affiliations:** Laboratoire CLLE, CNRS, University of Toulouse, Université Toulouse Jean-Jaurès, Toulouse, France

**Keywords:** COVID-19, lockdown, well-being, inner peace, psychological resources, positive expectancies, wisdom, gratitude

## Abstract

This longitudinal study investigated the capability of various positive psychological resources to directly or indirectly protect specific well-being outcomes and moderate the effects on well-being of health and economic threats in a lockdown situation during the 2020 health crisis in France. At the beginning of lockdown (wave 1), participants (*N* = *470*) completed self-assessment questionnaires to document their initial level of well-being and state of nine different well-established psychological resources, measured as traits: optimism, hope, self-efficacy, gratitude toward the world, self-transcendence, wisdom, gratitude of being, peaceful disengagement, and acceptance. Three weeks later, a weekly follow-up was started to record changes in well-being and reported threats for a duration of 5 weeks (waves 2–6). Results show that psychological resources efficiently protected well-being in a variety of ways: they buffered the adverse effects of reported threats to health and wealth, increased the well-being averages, and reduced the decline in well-being over time. More specifically, emotional well-being was positively predicted by hope, gratitude of being, and, to a lesser level, by acceptance; psychological well-being by self-efficacy, personal wisdom, and gratitude of being; social well-being only by gratitude toward the world; and inner well-being by optimism, gratitude of being, and acceptance. The study emphasizes the importance of cultivating psychological resources in ordinary times to protect individuals' well-being when difficult and extraordinary circumstances occur. It also offers clues to the kind of resources one may want to develop.

## Introduction

In January 2020, the new coronavirus (SARS-CoV-2) was identified as the cause of the COVID-19 disease that plagued the city of Wuhan, China (Zhou et al., 2020). The spread of the virus around the world was extremely rapid, to the point that the World Health Organization (WHO) declared it a pandemic and exhorted governments to act “aggressively” to contain the virus (World Health Organization, 2020). In fact, in many countries, authorities took more or less aggressive measures of quarantine, mass testing, mask enforcement, etc. In particular, many countries implemented “lockdown” as a response, leading half of the world's population (more than 3.9 billion people) to be instructed to stay home (Sandfor, 2020). The economic and political consequences of the situation were huge. Lockdown reduced social interactions. Mortality salience reached unusual levels in most modern countries. This very complex situation considerably affected the well-being of populations (e.g., Brodeur et al., 2020; Chen et al., 2020; Greyling et al., 2020).

When sudden crises arise, some factors that take time to change may have dramatic consequences. Obesity, for example, considerably increases the probability of a bad outcome if the person is infected (Dietz and Santos-Burgoa, 2020). On the psychological side, it is a reasonable hypothesis that some acquired dispositions can have protective effects on the ability to cope with stressful crises (e.g., Windle and Woods, 2004). Because these traits take time to acquire, they must be developed with anticipation, i.e., long before the occurrence of a crisis. This article presents the results of a longitudinal study that explored the potentially protective role of a range of psychological resources against the adverse effects of lockdown in a sample of French citizens.

### Well-Being During Pandemic and Lockdown

We will first detail how well-being is addressed in this article. We then turn to the question of how the unprecedented situation generated by Covid-19, including lockdowns throughout the world, could impacted well-being.

#### The Construct of Well-Being

The psychological study of well-being has been very active over the past 25 years (Linton et al., 2016) and has led to a plethora of approaches (Dodge et al., 2012). For this study, we selected two: the three-dimensional model of positive mental health (Keyes, 2002) and inner harmony (Dambrun et al., 2012; Delle Fave et al., 2016). Keyes's (2002) three-dimensional model combines emotional well-being (EWB), psychological well-being (PWB), and social well-being (SWB). These dimensions are grounded on the two main conceptualizations of well-being, both rooted in major philosophical traditions, namely, subjective (or “hedonic”) and psychological (or “eudaimonic”) well-being (Huta, 2017). Mainly attached to the hedonic tradition, subjective well-being is defined as a high level of positive affect, a low level of negative affect, and a high degree of satisfaction with one's life (Diener et al., 2009). In this approach, well-being is considered subjective in the sense that only individuals can assess their own wellness, and, here, the source of this happiness is not considered. In Keyes's (2002) model, subjective well-being is referred to as emotional well-being (EWB). In contrast, the eudaimonic tradition considers well-being as an optimal functioning through the endorsement of virtues and the actualization of one's potential. Psychological well-being (PWB) has been operationalized as the combination of self-acceptance, autonomy, purpose in life, positive relationships with others, environmental mastery, and personal growth (Ryff and Keyes, 1995). It was adapted as such in Keyes's (2002) model. Keyes (1998) developed an extension of PWB to Social Well-Being (SWB), which refers to the social dimension of the eudaimonic approach. SWB assesses positive social functioning through five dimensions: social coherence, social actualization, social integration, social acceptance, and social contribution. The three-factor structure, with EWB, PWB, and SWB, has demonstrated good internal and discriminant validity (Gallagher et al., 2009; Lamers et al., 2011; Joshanloo, 2016). In a cross-cultural study, inner-harmony—including peace of mind and tranquility feelings—has been the most widely reported lay definition of happiness (Delle Fave et al., 2016). We thus propose to complement the previous three dimensions with one that has long been forgotten in the scientific literature, which we will call here “inner well-being” (IWB). Inner well-being (IWB) can be understood as low arousal feelings of peace of mind, which are believed to be more stable and less dependent on external stimuli than high arousal positive feelings (Dambrun et al., 2012). Dambrun et al. (2012) describe IWB (i.e., “authentic-durable happiness” in their paper) as “an optimal way of being, a state of durable contentment and plenitude or inner-peace (…) based on a quality of consciousness that underlies and imbues each experience” (p. 2). If our theoretical approach that poses multiple dimensions to well-being is well-founded, we should expect to observe specific sets of resources correlating with the different dimensions.

#### Covid-19, Lockdowns, and Threats: Consequences for Well-Being

**Figure 1:**
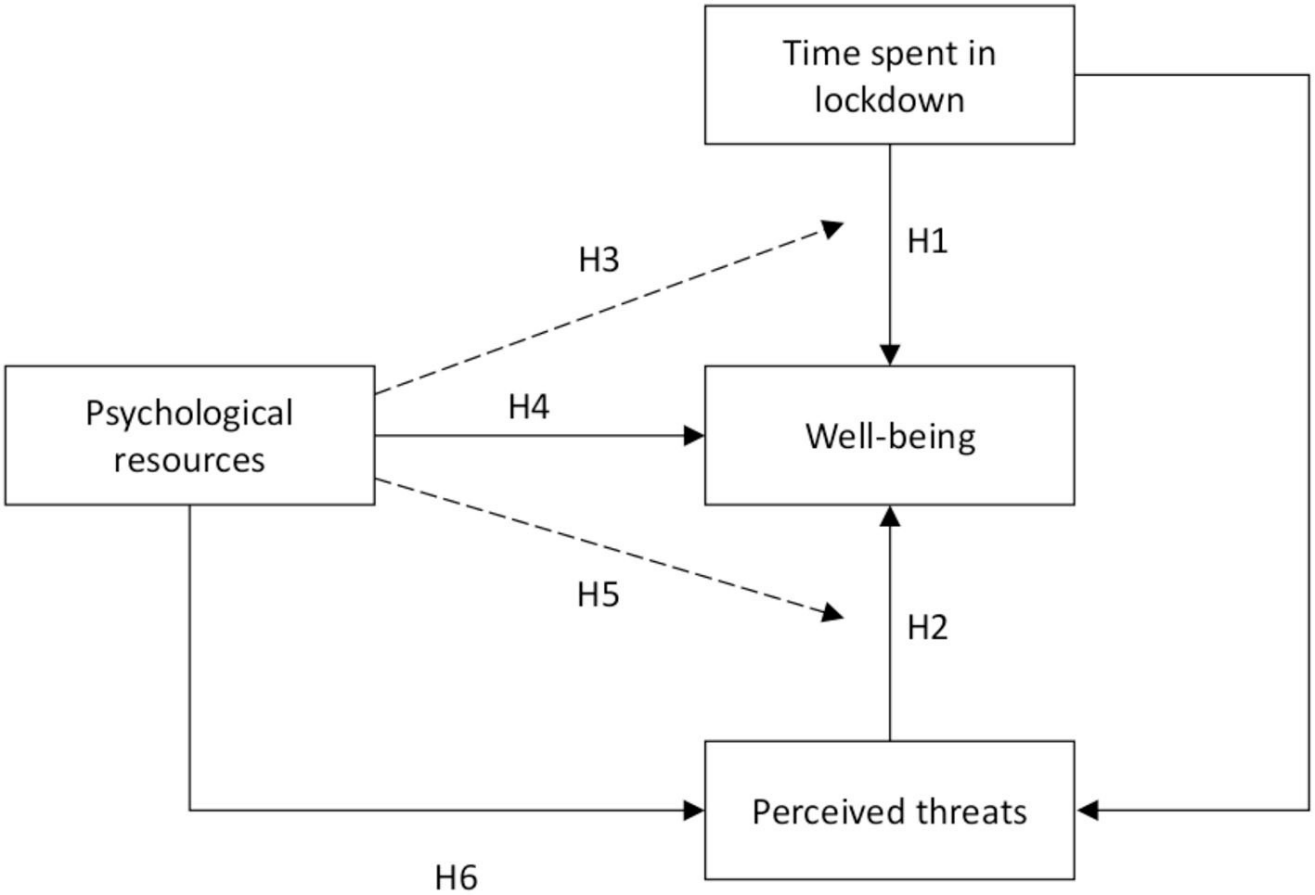
Hypotheses of the present study. Solid lines depict direct effects hypotheses, dashed lines depict moderation hypotheses.

We see several ways through which the lockdown, and more broadly the pandemic situation, can affect well-being. Figure 1 depicts the main hypotheses of this study. To begin with, we expected changes in well-being as time passed, which motivated a longitudinal study in the first place. However, this overly simple hypothesis calls for refinements. Perhaps the most intuitive hypothesis is a general and progressive reduction of well-being in the population under lockdown. Note that, as intuitive as it may appear, this hypothesis has not yet been tested using modern psychology tools because no other pandemic in the modern era has triggered such strong and extensive governmental measures. Feelings of loneliness (Steptoe et al., 2013) can be expected to increase in isolated persons during a lockdown. People confined together might see their relationships deteriorate as the lockdown progresses. People may also lose some of the social support they normally receive and see their well-being affected accordingly (Lincoln, 2000). On the other hand, renewing family ties by stopping school and work can be positive, at least initially when parents are not yet exhausted by their increasing responsibilities (Hubert and Aujoulat, 2018) and when children and adolescents do not yet suffer from being separated from their peers for long periods of time (Brown and Larson, 2009). Leisure activities have also been reported as an important correlate of well-being (Han and Patterson, 2007; Adams et al., 2010), and people in lockdown are likely prevented from engaging in them. All in all, we expected a decrease in WB over time, but we also expected that well-being would gradually rise back up to its chronic level either after the effective end of the lockdown or after the official announcement of this end (H1). This kind of return to some baseline level of well-being has since Brickman and Campbell (1971) long been documented in the literature. Despite debates on the determinants of the baseline (e.g., Lyubomirsky et al., 2005; Lucas, 2007), it is reasonable to say that happiness will return to around its initial value when lockdown is over, as most of the impairments to well-being will cease at the same time. It turned out that a few weeks after the study started, the French authorities announced the forthcoming end of lockdown. This event in itself could also trigger a partial recovery in well-being levels, even though we had not really anticipated its occurrence. The conjunction of a general downward trend in WB, followed by the ascent bound to the release of lockdown, led us to conjecture a U-shaped curve with an initial decrease in well-being followed by an increase that would gradually catch up around a set point.

**Hypothesis 1**: Well-being will decrease as the lockdown progresses and will tend to return to its initial level when the end of lockdown is near.

Obviously, the pandemic situation in general may increase the feeling of being threatened. People may fear for their health, sometimes even for their lives, but also for the health of their friends and family, especially those whose health is fragile or who have risk factors that increase their chances of developing severe respiratory problems when infected with the virus, such as the elderly (Wu et al., 2020). We believe that feeling health threats would be predictive of well-being: the higher the level of threat to personal and relatives' health is reported, the lower the level of well-being should be. The second threat that may be important to consider is the economic situation. Some people have had no choice but to close their businesses. Others have lost their jobs, partially or totally. This leads to uncertainty about financial matters and, therefore, to more stress and anxiety, as exemplified by the 2008 economic crisis (Deaton, 2011). In addition, as with the health threat, the economic threat to a friend, and especially a member of the family, can be a cause of distress. We have thus made the following hypothesis (see Figure 1):

**Hypothesis 2**: Economic and health threats will affect well-being. More precisely, the reported threat to health and to one's own economic situation and that of a close relative has a negative impact on one's well-being.

### Psychological Resources

Throughout history, catastrophic diseases have killed innumerable humans and compromised economic activities. It would be no surprise if strong psychological resources had been selected for dealing with such disasters. The main objective of this study was thus to test the putative protective role of psychological resources on well-being in a pandemic and lockdown context.

Hobfoll (2002) defined resources as “those entities that either are centrally valued in their own right (e.g., self-esteem, close attachments, health, and inner peace) or act as a means to obtain centrally valued ends (e.g., money, social support, and credit)” (p. 307). Speaking of protective psychological resources, we focus mainly on the second part of the definition of a resource, that is, all the mental dispositions and cognitive habits that are beneficial for well-being. We considered several routes through which psychological resources could have contributed to well-being during the lockdown (Figure 1). First, psychological resources can directly affect the level of well-being (H3). Also, as mentioned earlier, we expected that the different well-being outcomes would have different sets of resource predictors, thus validating their discriminant validity. Second, the temporal evolution of well-being during lockdown could be moderated so that people with high psychological resources would observe a smaller decrease in well-being or no decrease at all (H4). Third, psychological resources could buffer the effect of threats to well-being (H5). For example, self-efficacy could reduce the expected negative effect on well-being of economic threat. High self-efficacy would be associated with high confidence in the ability to cope with this threat. Fourth, we expected that psychological resources would decrease reported threat and then have a positive effect on well-being through this reduction (H6). In total, we had four hypotheses about how psychological resources affect well-being during the lockdown (see Figure 1):

**Hypothesis 3**: Psychological resources will directly affect well-being during the lockdown.

**Hypothesis 4**: Psychological resources will moderate the evolution of well-being during lockdown.

**Hypothesis 5**: Psychological resources will moderate health and economic threats during lockdown.

**Hypothesis 6**: Psychological resources will directly reduce the reported health and economic threats and indirectly increase well-being by the reduction of threat feelings.

To investigate which psychological resources might prevent the detrimental effects of a prolonged lockdown, we selected a set of psychological resources on the basis of three main criteria. First, the association of the resource with well-being as well as its protective effect against risk factors had to be theoretically grounded. Second, these relationships should have been previously confirmed by a large body of work using a rigorous scientific method. Third, the resource had to be measurable through a scale with good psychometric properties and, if possible, already validated in French. The psychological resources selected for the purpose of this study were: self-efficacy, optimism, hope, wisdom, gratitude toward the world, gratitude of being, peaceful disengagement, and acceptance. We briefly describe all of these resources and present research evidence of their contribution to well-being and their protective role against economic and health threats.

#### Dispositions Toward Positive Expectancy

*Self-efficacy*—i.e., people's beliefs about their capabilities to produce desired effects—is one of the most widely studied psychological resources in psychology (Bandura, 2010). When individuals believe that their actions can actually have a positive impact on the world, they are more likely to engage in such activities. Self-efficacy thus predicts the adoption of effective behaviors, so this should also lead to the satisfaction that accompanies the achievements obtained through these behaviors. Accordingly, it has been shown that self-efficacy predicts performance in the workplace (Stajkovic and Luthans, 1998), job satisfaction, and prevents job burnout (e.g., in teachers, Zee and Koomen, 2016). It also influences health-related intentions and behaviors (Sheeran et al., 2016), promotes medication adherence (Náfrádi et al., 2017), and serves as a protective variable in the experience of post-traumatic stress disorder, general distress, and somatic health (Luszczynska et al., 2009). Furthermore, people with high self-efficacy showed greater attentional bias toward well-being stimuli than toward threat-related stimuli (Karademas et al., 2007).

*Optimism* is a positive expectancy about future events. Dispositional optimism is an individual difference variable that determines to what degree people are generally optimistic about their lives (Carver et al., 2010). It has been consistently demonstrated in a wide variety of contexts that optimists are likely to experience more positive and less negative emotions than pessimistic people when faced with a difficult situation, including health problems (Carver et al., 2010). Moreover, optimistic people were physically healthier and attained higher job performance (Forgeard and Seligman, 2012). Finally, dispositional optimism has been positively associated with approach coping strategies and negatively associated with avoidance coping strategies (Nes and Segerstrom, 2006).

*Hope*, as defined by Snyder (2002), is the perceived capability to (a) derive pathways to desired goals (i.e., “pathway thinking”) and (b) motivate oneself to use those pathways (i.e., “agency thinking”). When treated as a trait, the variable has been associated positively with satisfaction with life, psychological well-being, and mental health. It has been negatively associated with psychopathological symptoms such as anxiety and depression (Delas et al., 2015). Hopeful people cope more effectively with stressful health-related situations (Kennedy et al., 2009). There is also strong evidence that hope predicts performance and well-being at work (Reichard et al., 2013).

#### Wisdom

Although consensus on the definition of wisdom is still lacking, wisdom researchers agree that it is mainly composed of metacognitive and self-transcendent abilities in combination with the motivation to work for the common good (Grossmann et al., 2020). This paper uses two wisdom models, the three-dimensional model of personal wisdom (Ardelt, 2003) and wisdom as self-transcendence (Levenson et al., 2005).

In the *Personal wisdom* approach, wisdom is understood as a personality trait of wise persons (Ferrari and Weststrate, 2013). One of the most prominent approaches to personal wisdom is the three-dimensional model that combines cognitive, reflective, and affective qualities (Ardelt et al., 2019). The cognitive dimension refers to the ability to understand life and the significance of phenomena. The affective dimension refers to the extent to which an individual feels compassionate care and concern for others. The reflective dimension captures how much one is engaged in a self-reflection aimed at reducing one's subjectivity and projections. For Ardelt (2003), all three dimensions must be present to speak of a “wise” person. Because wisdom helps individuals to adapt their behaviors to life's challenges and to accept difficult circumstances, it should be associated with better well-being in the long term (Ardelt, 2016). Measured as a three-dimensional personal quality, wisdom has indeed been associated with both subjective and psychological well-being, with stronger evidence and size effects for the latter (Ardelt, 2019).

*Self-transcendence (ST)* has been defined in a multitude of ways (Aldwin et al., 2019). One of the most prominent approaches is the liberative model (Levenson et al., 2005) in which self-transcendence is understood as “the ability to dissolve the rigid boundaries between the self and others” (Aldwin et al., 2019, p. 126), and to be the final stage of a development process (Curnow, 1999). This disposition to feel united with others is believed to have a positive impact on well-being by reducing the strong emotional reactions rooted in excessive self-interest (Bauer and Wayment, 2008; Dambrun and Ricard, 2011). Accordingly, ST was found to be positively correlated with various forms of well-being, including physical and emotional well-being, positive mental health, and positive emotions (Aldwin et al., 2019).

#### Gratitude Toward the World

Gratitude is a positive emotion that is experienced based on an appreciative orientation toward the world. Gratitude is believed to be beneficial to well-being due to its positive valence and its orientation toward prosociality and spirituality (McCullough et al., 2002; Wood et al., 2010). The disposition to be grateful toward the world is an affective trait characterized by the intensity and frequency of the experience of gratitude as well as the variety of life circumstances in which it is experienced (McCullough et al., 2002). It has been positively associated with a great range of well-being-related outcomes, such as positive and negative affects, life satisfaction, hedonic and eudemonic well-being, and depression (Wood et al., 2010). Experimental studies that evaluated interventions designed to increase gratitude drew similar conclusions, for example, in terms of how it improves well-being and alleviates depressive symptoms (Sin and Lyubomirsky, 2009). Wood et al. (2008) suggested that dispositional gratitude is part of a larger construct that includes all life orientations toward noticing and appreciating the positive in the world. Gratitude, as construed in this general approach, may be distinguished from the unconditional gratitude for the mere fact of being alive (Kan et al., 2009).

#### Minimalist Style: Gratitude of Being and Peaceful Disengagement

Kan et al. (2009) explored cultural differences in the centrality of well-being, comparing Eastern, and Western conceptions. They concluded that Eastern conceptions of happiness are “minimalist”, rooted in a view of the nothingness of things (i.e., nothing exists as absolute and permanent) and on the interdependent nature of the self (Kitayama et al., 2007). Their minimalist well-being scale included two aspects: gratitude of being concerns the appreciation of the mere fact of being and peaceful disengagement represents a peaceful attitude toward disengaging the self from reality. Gratitude of being is positively associated with markers of eudemonic well-being (self-acceptance, positive relations, purpose in life, and personal growth) and subjective well-being (life satisfaction and positive emotions). Peaceful disengagement, instead, is only correlated with self-acceptance, life satisfaction, and positive affect. Although in comparison to others there has been little interest in these resources in the literature, these two dimensions were included here for their potential relevance to the particular context of lockdown. When habitual activities and interactions are largely reduced or stopped altogether, it may be particularly advantageous to have the predispositions to be grateful for the simple fact of being and to peacefully disengage one's self from those activities.

#### Acceptance

The last resource selected in this study is the disposition to accept whatever happens. Acceptance is a mental attitude that allows non-reactivity in the present moment no matter the content of one's experience (Hayes et al., 2009; Lindsay and Creswell, 2017). It is an element in some emotional regulation strategy models (Garnefski et al., 2001). When facing a difficult life event that is not under control, acceptance can be a good strategy (Nakamura and Orth, 2005). Acceptance has been shown to reduce pain-related cognition in people experiencing chronic pain (Esteve et al., 2007; Chiros and O'Brien, 2011) and to improve the quality of life of patients having multiple sclerosis (Wilski et al., 2019) or incurable cancer (Nipp et al., 2016). The willingness to generally accept what is going on in one's life could be particularly helpful in the context of lockdowns over which people have little control.

## Methods

### Participants

We recruited the participants on a voluntary basis via an advertisement on social networks in France. A total of 674 participants fully completed the first wave and provided their email addresses in the questionnaire. Among them, 21 participants stated that they were living outside France, one was not of age and did not have parental authorization to participate, and six did not report their gender. They were therefore excluded. In the remaining pool of 646 participants, some took part in only one of the six waves^1^. We only kept individuals who responded to at least two waves. A total of 470 participants were thus included in the analyses. The demographics of these participants for each wave are presented in Table 1. After completing the final survey, all participants could ask for their “well-being curve”, which represented their score on each well-being variable on the waves they had responded to during the study.

**Table 1:** Sociodemographic characteristics of participants at each wave.

**Wave**	**1**	**2**	**3**	**4**	**5**	**6**
Total *N*	470	343	362	325	297	323
Gender						
Men	94 (20%)	74 (21.6%)	72 (19.9%)	56 (17.2%)	53 (17.8%)	49 (15.2%)
Women	376 (80%)	269 (78.4%)	290 (80.1%)	269 (82.8%)	244 (82.2%)	274 (84.8%)
Age M (SD)	42.7 (13.8)	42.4 (13.8)	43.0 (13.9)	43.7 (14.0)	44.4 (13.7)	44.5 (14.0)
15–25	62 (13.2%)	47 (13.7%)	46 (12.7%)	39 (12%)	29 (9.8%)	35 (10.8%)
25–35	94 (20%)	68 (19.8%)	73 (20.2%)	59 (18.2%)	56 (18.9%)	58 (18%)
35–45	111 (23.6%)	84 (24.5%)	79 (21.8%)	76 (23.4%)	66 (22.2%)	67 (20.7%)
45–55	106 (22.6%)	78 (22.7%)	89 (24.6%)	77 (23.7%)	80 (26.9%)	85 (26.3%)
55–65	74 (15.7%)	48 (14%)	56 (15.5%)	58 (17.8%)	49 (16.5%)	58 (18%)
65–82	23 (4.9%)	18 (5.2%)	19 (5.2%)	16 (4.9%)	17 (5.7%)	20 (6.2%)
Monthly income						
<1,000	86 (18.3%)	57 (16.6%)	59 (16.3%)	55 (16.9%)	47 (15.8%)	55 (17%)
1,000–2,000	147 (31.3%)	110 (32.1%)	115 (31.8%)	100 (30.8%)	96 (32.3%)	99 (30.7%)
2,000–3,000	110 (23.4%)	79 (23%)	87 (24%)	78 (24%)	66 (22.2%)	78 (24.1%)
>3,000	127 (27%)	97 (28.3%)	101 (27.9%)	92 (28.3%)	88 (29.6%)	91 (28.2%)

*Wave 0 corresponds to the initial measurement time; waves 1–5 are weekly follow-ups. Each participant responded to at least two waves (including wave 0). Monthly incomes are in euros*.

### Procedure

This study followed a longitudinal panel over 8 weeks, starting the second week of the French lockdown. It consisted of three phases containing six waves of observation.

- Phase 1: participants filled in the first survey (i.e., wave 1) containing demographics and control variables as well as measures of interest for psychological resources and well-being.
- Phase 2: 3 weeks after Phase 1, volunteers were contacted via email to complete a series of four short weekly surveys (i.e., waves 2–5), including well-being and threat measures. Some measures unrelated to this article were also taken (e.g., activities).
- Phase 3: the final survey (i.e., wave 6) happened 1 week after phase 2, just after the end of lockdown in France, and contained the same threats and well-being variables as wave 1.

### Materials

#### Psychological Resources

All psychological resources were uniformly surveyed using a Likert scale ranging from 1 = “strongly disagree” to 7 = “strongly agree,” except for Acceptance.

##### Hope, Optimism, and Self-efficacy

We assessed hope (e.g., “If I should find myself in a jam, I could think of many ways to get out of it”), optimism (e.g., “I am looking forward to the life ahead of me”), and self-efficacy (e.g., “I am confident that I could deal efficiently with unexpected events”) using the Compound-Psychological-Capital questionnaire (CPC-12, Lorenz et al., 2016). Reliabilities were satisfactory for hope (α = 0.79), optimism (α = 0.84), and self-efficacy (α = 0.79).

##### Personal Wisdom

Personal wisdom was assessed with the 12-Item Abbreviated Three-Dimensional Wisdom Scale (3D-WS-12, Thomas et al., 2017), which uses four items to measure each of three dimensions of wisdom, as theorized by Ardelt (2003): cognitive (e.g., “A problem has little attraction for me if I don't think it has a solution”), affective (e.g., “Sometimes I feel a real compassion for everyone”), and reflective (e.g., “When I am confused by a problem, one of the first things I do is survey the situation and consider all the relevant pieces of information”). The personal wisdom measure was marginally reliable (α = 0.61.)

##### Self-transcendent Wisdom

Self-transcendent wisdom was assessed using the most recently published version of the Adult Self-Transcendence Inventory (ASTI, Koller et al., 2017). The classical version of the ASTI measured self-transcendence as a single dimension (Levenson et al., 2005). Koller et al. (2017) used a mixed-method procedure to assess the ASTI dimensionality, including item evaluations by wisdom and psychometric experts and quantitative analysis using Item Response Theory. They found five non-overlapping dimensions: “self-knowledge and integration,” “peace of mind,” “non-attachment,” “self-transcendence,” and “presence in the here-and-now and growth.” We selected all seven items of the dimension of self-transcendence as a measure of self-transcendent wisdom (e.g., “I feel that my individual life is part of a greater whole”, α = 0.81).

##### Gratitude Toward the World

The French version of the six-item Gratitude Questionnaire (GQ-6) was used to assess dispositional gratitude (McCullough et al., 2002; Shankland and Martin-Krumm, 2012) (e.g., “I have so much in life to be thankful for,” or “I am grateful to a wide variety of people”). This measure had adequate reliability in our sample (α = 0.79).

##### Gratitude for Being and Peaceful Disengagement

We used the Minimalist Well-Being Scale to assess gratitude for being and peaceful disengagement (Kan et al., 2009). Four items captured the disposition to be grateful for just being (e.g., “I feel grateful that I am alive”), and seven items captured peaceful disengagement (e.g., “It feels good to do nothing and relax”). Both construct reliabilities were satisfactory (gratitude for being: α = 0.87; peaceful disengagement: α = 0.77).

##### Acceptance

We used the eight items of the Acceptance dimension of the Brief Serenity Scale to assess the disposition to accept whatever happened (e.g., “I accept situations that I cannot change,” Kreitzer et al., 2009). We used the original Likert scale that assesses the frequency of the experience (1 = “never” to 5 = “always”). The measure was adequately reliable (α = 0.82).

#### Well-Being

We assessed well-being using two different tools: the Mental Health Continuum and Inner Peace. Most well-being variables were assessed using the French Canadian version of the Mental Health Continuum Short-Form (MHC-SF, Lamers et al., 2011; Doré et al., 2017). In each of 14 items, respondents report how frequently they have felt a certain way during the past month. The 6-point Likert scales range from 1 (“never”) to 6 (“always”). The MHC-SF items are grouped into three dimensions. The emotional well-being dimension (EWB) assesses positive emotions and satisfaction with life (e.g., “…how often did you feel happy”). The psychological and social well-being dimensions (PWB and SWB) assess eudaimonic well-being at the personal (e.g., “…how often did you feel that you liked most parts of your personality”) and social levels (e.g., “…how often did you feel that you had something important to contribute to society”). In wave 1, we adapted the instructions, replacing “during the past month” with “during the lockdown.” For the remaining waves, in order to be able to capture shorter fluctuations, the instructions referred to “the previous week.” To assess inner well-being (IWB), we used the five items of the inner peace dimension of the Subjective Authentic-Durable Happiness Scale (SA-DHS, Dambrun et al., 2012) (e.g., “…how often did you feel peace of mind”). All well-being measures had good reliability (α_*EWB*_ = 0.88; α_*PWB*_ = 0.80; α_*SWB*_ = 0.79; α_*IWB*_ = 0.95).

#### Reported Threats

In this article, we refer to “threats” as self-evaluations of threat provided by a participant. Even though threat assessments may depend on some objective features of their environment, participants always construct a subjective representation of the facts when judging, deciding (Kahneman and Tversky, 1979), or perceiving risks (Slovic et al., 2005). Actual income reduction, or job loss, or other types of objective data were not measured here. They remain outside the scope of this paper, even though some of them may have been present in participants' minds when they answered.

Threats were considered in terms of two domains: health and economic situation. The first domain, health, was addressed through three items. In the first item, participants answered to “Do you feel exposed to contamination from the virus?” using a five-point Likert scale (1 = “Absolutely not”, 2 = “Low exposure”, 3 = “Maybe or maybe not”, 4 = “Yes, quite exposed” and 5 = “Yes, absolutely”). The two other items investigated the degree to which respondents felt a threat to health regarding themselves (“To what degree do you feel your personal health is threatened by the epidemic?”) and their relatives (“To what degree do you feel the health of your relatives is threatened by the epidemic?”). The 5-point Likert scale ranged from 1 (“Probably no risk”) to 5 (“Very seriously threatened”).

Two other items concerned economic threat and were constructed in the same way as the last two items for reported health threat (“Is your economic situation threatened by the epidemic and the lockdown situation?” and “Is the economic situation of your relatives threatened by the epidemic and the lockdown situation?”).

### Data Analysis

We used R (Version 4.0.2; R Core Team, 2020) for all our analyses. All data and analyses can be found in an open repository of the Open Science Framework website: https://osf.io/45aq3. In order to account for the longitudinal nature of the data, we tested our hypotheses using linear mixed models with the *lmer* function of the *lme4* package (Bates et al., 2015). Sample sizes in each wave are presented in Table 1. The data from the 470 selected participants were used in all analyses. Intercepts were the only random parameters in all models (models that included “time” as a random parameter did not converge). Reported economic and health threat variables were time-dependent, that is, they were measured in each wave of the study. We computed the intra-class correlations for time-dependent variables (including the outcomes) using the *ICCbare* function of the *ICC* package (Wolak et al., 2012). ICC values ranged from 0.79 to 0.84. All psychological resource variables were time-invariant and were measured at wave 1. Well-being baseline (from wave 1), age, gender, and income were included as control time-invariant variables. To facilitate the estimation of models and the interpretation of results, all numerical variables—outcomes and predictors—were standardized: means were set to 0 and standard deviations to 1. Only time, which was coded by the number of weeks since the beginning of the lockdown, was left unstandardized. For all models, we provide marginal *R*^2^ (the proportion of variance explained by the fixed effects only) and conditional *R*^2^ (the proportion of variance explained by both fixed and random effects). Since the nine resource variables were moderately to highly correlated, we provide “zero-order” effects for individual resources and interactions. Zero-order effects were calculated from alternative models in which all other resources and interactions were not included as predictors. In addition, high multicollinearity between predictors is usually diagnosed by variance inflation factors (VIF) >5 (O'brien, 2007). In this study, VIF were computed using the *vif* function of the *car* package (Fox and Weisberg, 2019). No VIF exceeded 2.51.

To test H1–H4, fixed effects were estimated in three steps for each happiness variable. In step 1, reported threats along with the time spent since lockdown (in weeks) were estimated first, with an additional second-level quadratic effect of time in order to model the expected U-shaped curve (H1 & H2). In step 2, all psychological-resource variables were simultaneously added to the model (H3). In step 3, two-way interactions between psychological resources, reported threats, and time spent since lockdown were estimated simultaneously (H4 & H5).

In order to test H6, two models with each reported threat as a dependent variable were estimated, with time spent in lockdown and all psychological resources modeled as fixed effects. We also tested the indirect effects of psychological resources on well-being through reported threats using the *mediate* function of the *mediation* package (Tingley et al., 2014), which also provided confidence intervals by quasi-Bayesian approximation.

## Results

Table 2 presents the means and standard deviations for all scores. It also reports pairwise correlations between scores at wave 1 and intra-class correlations for longitudinal variables. A Bonferroni correction for 105 comparisons was applied to the *p*-values of the correlation matrix between the 15 variables.

**Table 2:** Descriptive statistics and correlation matrix of the study variables as measured in wave 1.

	***M***	***SD***	**ICC**	**1**	**2**	**3**	**4**	**5**	**6**	**7**	**8**	**9**	**10**	**11**	**12**	**13**	**14**
1. EWB	4.14	1.07	0.79	–													
2. PWB	4.49	0.91	0.84	0.65^***^	–												
3. SWB	3.10	0.94	0.83	0.54^***^	0.62^***^	–											
4. IWB	4.29	1.35	0.77	0.62^***^	0.58^***^	0.39^***^	–										
5. H-threat	1.88	0.71	0.65	−0.14	−0.05	−0.02	−0.19^**^	–									
6. E-threat	1.59	0.88	0.80	−0.12	−0.01	0.01	−0.16	0.17^*^	–								
7. Optimism	4.84	1.31	–	0.51^***^	0.48^***^	0.37^***^	0.45^***^	−0.16	−0.10	–							
8. Self-efficacy	5.43	0.96	–	0.40^***^	0.51^***^	0.32^***^	0.45^***^	−0.11	0.02	0.45^***^	–						
9. Hope	5.04	1.10	–	0.50^***^	0.55^***^	0.37^***^	0.48^***^	−0.09	−0.12	0.58^***^	0.60^***^	–					
10. P-Wisdom	4.00	0.72	–	0.38^***^	0.50^***^	0.36^***^	0.47^***^	−0.03	−0.03	0.33^***^	0.48^***^	0.45^***^	–				
11. ST-Wisdom	4.97	1.07	–	0.33^***^	0.39^***^	0.31^***^	0.33^***^	−0.02	0.03	0.36^***^	0.35^***^	0.32^***^	0.38^***^	–			
12. Grat-world	4.73	0.99	–	0.54^***^	0.52^***^	0.53^***^	0.48^***^	−0.02	−0.04	0.52^***^	0.33^***^	0.47^***^	0.44^***^	0.50^***^	–		
13. Grat-being	5.39	1.34	–	0.63^***^	0.53^***^	0.44^***^	0.53^***^	−0.02	−0.11	0.57^***^	0.32^***^	0.43^***^	0.33^***^	0.41^***^	0.64^***^	–	
14. PD	5.21	0.93	–	0.46^***^	0.35^***^	0.19^**^	0.45^***^	−0.11	−0.04	0.35^***^	0.35^***^	0.39^***^	0.22^***^	0.33^***^	0.38^***^	0.43^***^	–
15. Acc	3.44	0.62	–	0.52^***^	0.57^***^	0.39^***^	0.60^***^	−0.10	−0.02	0.42^***^	0.49^***^	0.46^***^	0.51^***^	0.50^***^	0.51^***^	0.50^***^	0.49^***^

ICC, intra-class correlations for time-dependent variables; EWB, emotional well-being; PWB, psychological well-being; SWB, social well-being; IWB, inner well-being; ST-Wisdom, self-transcendent wisdom; P-Wisdom, personal wisdom; Grat-world, gratitude toward the world; Grat-being, gratitude of being; PD, peaceful disengagement; Acc, acceptance.

* p < 0.05;

** p < 0.01;

*** *p < 0.001 after Bonferroni correction for 105 comparisons*.

**Figure 2:**
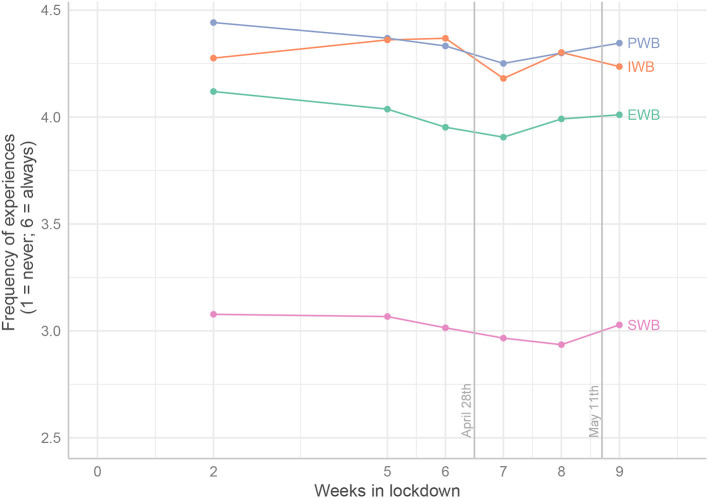
Slopes of all well-being observed means from the first assessment to the end of the survey. EWB, emotional well-being; PWB, psychological well-being; SWB, social well-being; IWB, inner well-being. The vertical lines display important events: April 28, announcement of the date of the end of the lockdown; May 11, end of the French lockdown.

Figure 2 shows the time-dependent pattern of well-being observed means during and after the French lockdown. Two vertical axes exhibit important events: (1) on April 28, Prime Minister Édouard Philippe announced the probable end of lockdown by May 11, provided certain conditions were met; and (2) on May 11, people actually got out of lockdown.

Results of the three-steps models are presented in Tables 3–6 for each dependent variable, namely EWB, PWB, SWB, and IWB. No control variables (i.e., age, gender, and annual income) significantly affected any of the WB variables. We can note that the VIF never exceeded 3. We can therefore assume that multicollinearity is not an issue. Nevertheless, for each resource and interaction, the zero-order effect—that is when other resources are not included in the model—is displayed in the tables.

**Table 3:** Step by step standardized estimates of the effects of time and reported threat (Step 1), psychological resources (Step 2), and their interactions (Step 3) on Emotional Well-Being.

	**Step 1**	**Step 2**	**Step 3**	**VIF**	**Zero-order**
Intercept	0.07 [−0.23; 0.38]	0.10 [−0.18; 0.38]	0.10 [−0.18; 0.39]		
Time	−0.73 [−1.75; 0.29]	−0.78 [−1.79; 0.24]	−1.11^*^ [−2.15; −0.06]	1.22	
Time^2^	1.03^*^ [0.06; 2.00]	1.04^*^ [0.08; 2.01]	0.55 [−0.44; 1.55]	1.12	
E-threat	−0.07^**^ [−0.11; −0.02]	−0.06^**^ [−0.10; −0.02]	−0.05^*^ [−0.10; −0.01]	1.14	
H-threat	−0.04 [−0.08; 0.00]	−0.04. [−0.08; 0.00]	−0.04^*^ [−0.08; 0.00]	1.29	
Age	0.03 [−0.03; 0.09]	0.00 [−0.06; 0.07]	0.00 [−0.06; 0.06]	1.29	
Gender-women	−0.06 [−0.22; 0.09]	−0.05 [−0.19; 0.09]	−0.05 [−0.19; 0.09]	1.07	
Income-low	0.05 [−0.13; 0.23]	−0.03 [−0.19; 0.14]	−0.04 [−0.21; 0.13]	1.26	
Income-medium	0.07 [−0.13; 0.26]	0.02 [−0.16; 0.20]	0.02 [−0.17; 0.20]		
Income-high	0.07 [−0.12; 0.26]	0.00 [−0.17; 0.18]	0.01 [−0.17; 0.18]		
EWB-baseline	0.63^***^ [0.57; 0.69]	0.38^***^ [0.31; 0.46]	0.38^***^ [0.30; 0.46]	2.12	
Optimism		0.03 [−0.05; 0.11]	0.03 [−0.05; 0.11]	2.19	0.20^***^
Self-efficacy		0.04 [−0.03; 0.12]	0.05 [−0.03; 0.12]	1.98	0.16^***^
Hope		0.14^***^ [0.06; 0.22]	0.14^***^ [0.06; 0.22]	2.33	0.22^***^
ST-Wisdom		−0.05 [−0.12; 0.02]	−0.05 [−0.12; 0.02]	1.64	0.14^***^
P-Wisdom		0.03 [−0.04; 0.10]	0.04 [−0.04; 0.11]	1.75	0.08^*^
Grat-world		−0.01 [−0.09; 0.07]	−0.01 [−0.09; 0.07]	2.41	0.25^***^
Grat-being		0.20^***^ [0.12; 0.29]	0.21^***^ [0.12; 0.29]	2.47	0.15^***^
PD		−0.06 [−0.12; 0.01]	−0.07 [−0.14; 0.00]	1.61	0.05
Acc		0.11^**^ [0.03; 0.19]	0.11^**^ [0.03; 0.19]	2.24	0.20^***^
Time × H-threat			−1.39^**^ [−2.45; −0.34]	1.17	
H-threat × Self efficacy			0.05^*^ [0.00; 0.11]	2.48	0.03
Time × PD			−2.13^***^ [−3.36; −0.90]	2.42	−1.47^**^
Marginal *R*^2^	0.43	0.50	0.50		
Conditional *R*^2^	0.79	0.79	0.79		

95% confidence intervals in brackets. Only significant interactions are presented in Step 3. The column “Zero-order” displays unstandardized betas of resource predictors and their interactions, when all other resources are not included in the model. Reference level for income estimates: very low income (<1,000 euros per month). EWB-baseline, baseline level of the well-being outcome measured at wave 1; ST-Wisdom, self-transcendent wisdom; P-Wisdom, personal wisdom; Grat-world, gratitude toward the world; Grat-being, gratitude of being; PD, peaceful disengagement; Acc, acceptance.

* p < 0.05;

** p < 0.01;

*** *p < 0.001*.

**Table 4:** Step by step standardized estimates of the effects of time and reported threat (Step 1), psychological resources (Step 2), and their interactions (Step 3) on Psychological Well-Being.

	**Step 1**	**Step 2**	**Step 3**	**VIF**	**Zero-order**
Intercept	−0.16 [−0.42; 0.11]	−0.15 [−0.40; 0.10]	−0.15 [−0.40; 0.11]		
Time	−2.00^***^ [−2.87; −1.12]	−1.99^***^ [−2.87; −1.12]	−2.06^***^ [−2.97; −1.16]	1.22	
Time^2^	1.36^**^ [0.52; 2.19]	1.42^***^ [0.59; 2.25]	1.26^**^ [0.40; 2.12]	1.12	
E-threat	−0.04^*^ [−0.08; 0.00]	−0.03 [−0.07; 0.01]	−0.03 [−0.06; 0.01]	1.14	
H-threat	−0.06^**^ [−0.09; −0.02]	−0.05^**^ [−0.08; −0.01]	−0.05^**^ [−0.08; −0.01]	1.29	
Age	0.00 [−0.05; 0.05]	0.02 [−0.04; 0.07]	0.01 [−0.04; 0.07]	1.33	
Gender-women	0.05 [−0.09; 0.18]	0.08 [−0.05; 0.20]	0.07 [−0.05; 0.20]	1.08	
Income-low	0.09 [−0.06; 0.25]	0.02 [−0.12; 0.17]	0.01 [−0.14; 0.16]	1.26	
Income-medium	0.14 [−0.03; 0.30]	0.07 [−0.08; 0.23]	0.08 [−0.08; 0.24]		
Income-high	0.06 [−0.10; 0.22]	−0.02 [−0.17; 0.14]	−0.02 [−0.17; 0.14]		
PWB-baseline	0.74^***^ [0.69; 0.80]	0.51^***^ [0.44; 0.58]	0.51^***^ [0.44; 0.58]	2.27	
Optimism		0.04 [−0.03; 0.11]	0.04 [−0.03; 0.11]	2.20	0.17^***^
Self-efficacy		0.08^*^ [0.01; 0.15]	0.08^*^ [0.01; 0.15]	2.00	0.17^***^
Hope		0.06 [−0.01; 0.13]	0.08^*^ [0.01; 0.15]	2.39	0.17^***^
ST-Wisdom		0.00 [−0.06; 0.06]	0.00 [−0.06; 0.06]	1.63	0.16^***^
P-Wisdom		0.09^**^ [0.03; 0.15]	0.09^**^ [0.02; 0.15]	1.77	0.10^***^
Grat-world		0.00 [−0.07; 0.07]	0.00 [−0.07; 0.07]	2.41	0.18^***^
Grat-being		0.12^***^ [0.05; 0.19]	0.11^**^ [0.04; 0.19]	2.32	0.13^***^
PD		0.02 [−0.04; 0.08]	0.01 [−0.05; 0.07]	1.58	0.11^***^
Acc		0.03 [−0.04; 0.10]	0.04 [−0.04; 0.11]	2.26	0.16^***^
E-threat × P Wisdom			0.06^*^ [0.01; 0.10]	1.67	0.04^*^
E-threat × PD			0.07^**^ [0.03; 0.11]	1.54	0.05^**^
H-threat × Optimism			−0.07^**^ [−0.11; −0.02]	2.22	−0.03
H-threat × Self efficacy			0.06^*^ [0.01; 0.10]	2.06	0.02
Time × PD			−1.34^*^ [−2.41; −0.28]	1.63	−0.80
Marginal *R*^2^	0.58	0.63	0.63		
Conditional *R*^2^	0.84	0.84	0.85		

95% confidence intervals in brackets. Only significant interactions are presented in Step 3. The column “Zero-order” displays unstandardized betas of resource predictors and their interactions, when all other resources are not included in the model. Reference level for income estimates: very low income (<1,000 euros per month). PWB-baseline, baseline level of the well-being outcome measured at wave 1; ST-Wisdom, self-transcendent Wisdom; P-Wisdom, personal Wisdom; Grat-world, Gratitude toward the world; Grat-being, gratitude of being; PD, peaceful disengagement; Acc, acceptance.

* p < 0.05;

** p < 0.01;

*** *p < 0.001*.

**Table 5:** Step by step standardized estimates of the effects of time and reported threat (Step 1), psychological resources (Step 2), and their interactions (Step 3) on Social Well-Being.

	**Step 1**	**Step 2**	**Step 3**	**VIF**	**Zero-order**
Intercept	−0.02 [−0.30; 0.27]	0.12 [−0.16; 0.40]	0.14 [−0.14; 0.43]		
Time	−2.02^***^ [−2.93; −1.11]	−1.98^***^ [−2.89; −1.08]	−2.19^***^ [−3.12; −1.25]	1.22	
Time^2^	1.11^*^ [0.25; 1.98]	1.14^**^ [0.28; 2.01]	0.65 [−0.23; 1.54]	1.12	
E-threat	−0.04^*^ [−0.08; 0.00]	−0.03 [−0.07; 0.01]	−0.02 [−0.06; 0.02]	1.13	
H-threat	−0.04^*^ [−0.07; 0.00]	−0.03 [−0.07; 0.01]	−0.03. [−0.07; 0.00]	1.29	
Age	0.01 [−0.05; 0.07]	0.03 [−0.03; 0.09]	0.03 [−0.03; 0.09]	1.31	
Gender-women	0.00 [−0.14; 0.14]	−0.04 [−0.18; 0.09]	−0.05 [−0.19; 0.09]	1.07	
Income-low	0.07 [−0.10; 0.24]	−0.02 [−0.19; 0.14]	−0.04 [−0.20; 0.13]	1.27	
Income-medium	−0.01 [−0.19; 0.18]	−0.04 [−0.21; 0.14]	−0.05 [−0.23; 0.13]		
Income-high	0.00 [−0.18; 0.18]	−0.05 [−0.22; 0.12]	−0.06 [−0.24; 0.11]		
SWB-baseline	0.70^***^ [0.64; 0.76]	0.55^***^ [0.49; 0.62]	0.55^***^ [0.48; 0.62]	1.65	
Optimism		0.07 [0.00; 0.15]	0.08^*^ [0.00; 0.16]	2.18	0.16^***^
Self-efficacy		−0.03 [−0.11; 0.04]	−0.03 [−0.11; 0.04]	1.97	0.07^*^
Hope		0.04 [−0.03; 0.12]	0.05 [−0.03; 0.13]	2.30	0.12^***^
ST-Wisdom		0.04 [−0.03; 0.11]	0.05 [−0.02; 0.12]	1.62	0.12^***^
P-Wisdom		0.05 [−0.02; 0.12]	0.04 [−0.03; 0.11]	1.74	0.12^***^
Grat-world		0.10^*^ [0.02; 0.18]	0.09^*^ [0.01; 0.18]	2.61	0.16^***^
Grat-being		0.06 [−0.01; 0.14]	0.06 [−0.02; 0.14]	2.24	0.20^***^
PD		−0.05 [−0.12; 0.01]	−0.07^*^ [−0.14; 0.00]	1.59	0.05
Acc		0.01 [−0.07; 0.09]	0.01 [−0.07; 0.09]	2.19	0.12^***^
Time × E-threat			1.03^*^ [0.07; 1.98]	1.18	
Time × H-threat			−1.33^**^ [−2.27; −0.39]	1.25	
E-threat × Self efficacy			−0.06^*^ [−0.11; 0.00]	2.21	−0.01
E-threat × PD			0.06^**^ [0.02; 0.10]	1.53	0.04^*^
Time × Grat-world			−1.70^*^ [−3.04; −0.36]	2.42	−0.81
Time × Grat-being			1.52^*^ [0.15; 2.88]	2.51	0.14
Time^2^ × Grat-being			1.80^**^ [0.52; 3.08]	2.23	0.85
Time × PD			−1.80^**^ [−2.90; −0.70]	1.64	−1.16^**^
Marginal *R*^2^	0.50	0.55	0.55		
Conditional *R*^2^	0.83	0.83	0.84		

95% confidence intervals in brackets. Only significant interactions are presented in Step 3. The column “Zero-order” displays unstandardized betas of resource predictors and their interactions, when all other resources are not included in the model. Reference level for income estimates: very low income (<1,000 euros per month). SWB-baseline, baseline level of the well-being outcome measured at wave 1; ST-Wisdom, self-transcendent wisdom; P-Wisdom, personal wisdom; Grat-world, gratitude toward the world; Grat-being, gratitude of being; PD, peaceful disengagement; Acc, acceptance.

* p < 0.05;

** p < 0.01;

*** *p < 0.001*.

**Table 6:** Step by step standardized estimates of the effects of time and reported threat (Step 1), psychological resources (Step 2), and their interactions (Step 3) on Inner Well-Being.

	**Step 1**	**Step 2**	**Step 3**	**VIF**	**Zero-order**
Intercept	0.25 [−0.06; 0.55]	0.30^*^ [0.00; 0.60]	0.32^*^ [0.01; 0.62]		
Time	−2.08^***^ [−3.13; −1.02]	−2.10^***^ [−3.15; −1.05]	−2.58^***^ [−3.66; −1.50]	1.22	
Time^2^	0.34 [−0.67; 1.34]	0.34 [−0.66; 1.34]	−0.25 [−1.27; 0.78]	1.12	
E-threat	−0.07^**^ [−0.12; −0.02]	−0.08^***^ [−0.13; −0.04]	−0.08^***^ [−0.13; −0.04]	1.15	
H-threat	−0.07^***^ [−0.12; −0.03]	−0.07^**^ [−0.11; −0.03]	−0.07^***^ [−0.12; −0.03]	1.29	
Age	0.01 [−0.05; 0.07]	0.03 [−0.04; 0.09]	0.03 [−0.04; 0.09]	1.29	
Gender-women	−0.14 [−0.29; 0.01]	−0.16^*^ [−0.31; −0.02]	−0.18^*^ [−0.33; −0.03]	1.07	
Income-low	0.05 [−0.13; 0.23]	0.04 [−0.14; 0.22]	0.03 [−0.15; 0.21]	1.25	
Income-medium	0.03 [−0.16; 0.22]	0.03 [−0.16; 0.22]	0.02 [−0.17; 0.21]		
Income-high	−0.05 [−0.24; 0.14]	−0.08 [−0.26; 0.11]	−0.06 [−0.25; 0.13]		
IWB-baseline	0.61^***^ [0.55; 0.67]	0.45^***^ [0.37; 0.53]	0.45^***^ [0.37; 0.53]	2.05	
Optimism		0.11^**^ [0.03; 0.19]	0.11^**^ [0.03; 0.20]	2.19	0.17^***^
Self-efficacy		0.07 [−0.01; 0.14]	0.07 [−0.01; 0.15]	1.98	0.11^***^
Hope		−0.02 [−0.10; 0.07]	−0.02 [−0.10; 0.07]	2.31	0.09^**^
ST-Wisdom		−0.02 [−0.10; 0.05]	−0.02 [−0.09; 0.05]	1.64	0.03
P-Wisdom		−0.06 [−0.14; 0.02]	−0.07 [−0.15; 0.01]	1.78	0.07^*^
Grat-world		0.00 [−0.08; 0.09]	0.02 [−0.07; 0.10]	2.40	0.14^***^
Grat-being		0.07 [−0.01; 0.16]	0.07 [−0.02; 0.15]	2.31	0.10^**^
PD		−0.02 [−0.09; 0.05]	−0.03 [−0.10; 0.04]	1.61	0.06
Acc		0.15^**^ [0.06; 0.23]	0.15^***^ [0.06; 0.24]	2.35	0.18^***^
Time × H-threat			−1.30^*^ [−2.38; −0.21]	1.25	
E-threat × Optimism			0.08^**^ [0.02; 0.14]	2.29	0.02
E-threat × Self efficacy			−0.09^**^ [−0.15; −0.03]	2.19	−0.05^*^
H-threat × Self efficacy			0.09^***^ [0.04; 0.15]	2.06	−0.02
Time × Self-efficacy			2.20^**^ [0.72; 3.69]	2.33	1.01^*^
Time^2^ × Grat-being			1.68^*^ [0.20; 3.16]	2.23	0.54
Time × PD			−2.27^***^ [−3.53; −1.00]	1.63	−1.02^*^
Marginal *R*^2^	0.42	0.45	0.47		
Conditional *R*^2^	0.77	0.77	0.79		

95% confidence intervals in brackets. Only significant interactions are presented in Step 3. The column “Zero-order” displays unstandardized betas of resource predictors and their interactions, when all other resources are not included in the model. Reference level for income estimates: very low income (<1,000 euros per month). IWB-baseline, baseline level of the well-being outcome measured at wave 1; ST-Wisdom, self-transcendent Wisdom; P-Wisdom, personal wisdom; Grat-world, gratitude toward the world; Grat-being, gratitude of being; PD, peaceful disengagement; Acc, acceptance.

* p < 0.05;

** p < 0.01;

*** *p < 0.001*.

### Overall Effects of Time Under Lockdown on Well-Being (H1)

EWB, PWB, SWB were all significantly negatively affected by time (in weeks) for the linear component (Tables 3–6, Step 1 column). The quadratic component (noted “Time^2^”) tended to be positive, which gives U-shaped curves (Figure 2). The exception was IWB, which had no significant quadratic effect. These results are consistent with H1.

When did the initial negative trend reverse? PWB and EWB attained their lowest values in the 6th week after the onset of lockdown, then the curve rose during the last 2 weeks. Interestingly, SWB reached a minimum only 1 week later. This general pattern shows that the official announcement of a precise date for the end of lockdown was a powerful enhancer for well-being.

### Effects of Economic and Health Threats on Well-Being (H2)

As expected (H2), economic and health reported threats affected all well-being variables, with the exception of EWB, which was not affected by reported threat to health (see Tables 3–6, Step 1 columns).

#### The Effects of Threats Changed With the Time Spent in Lockdown

**Figure 3:**
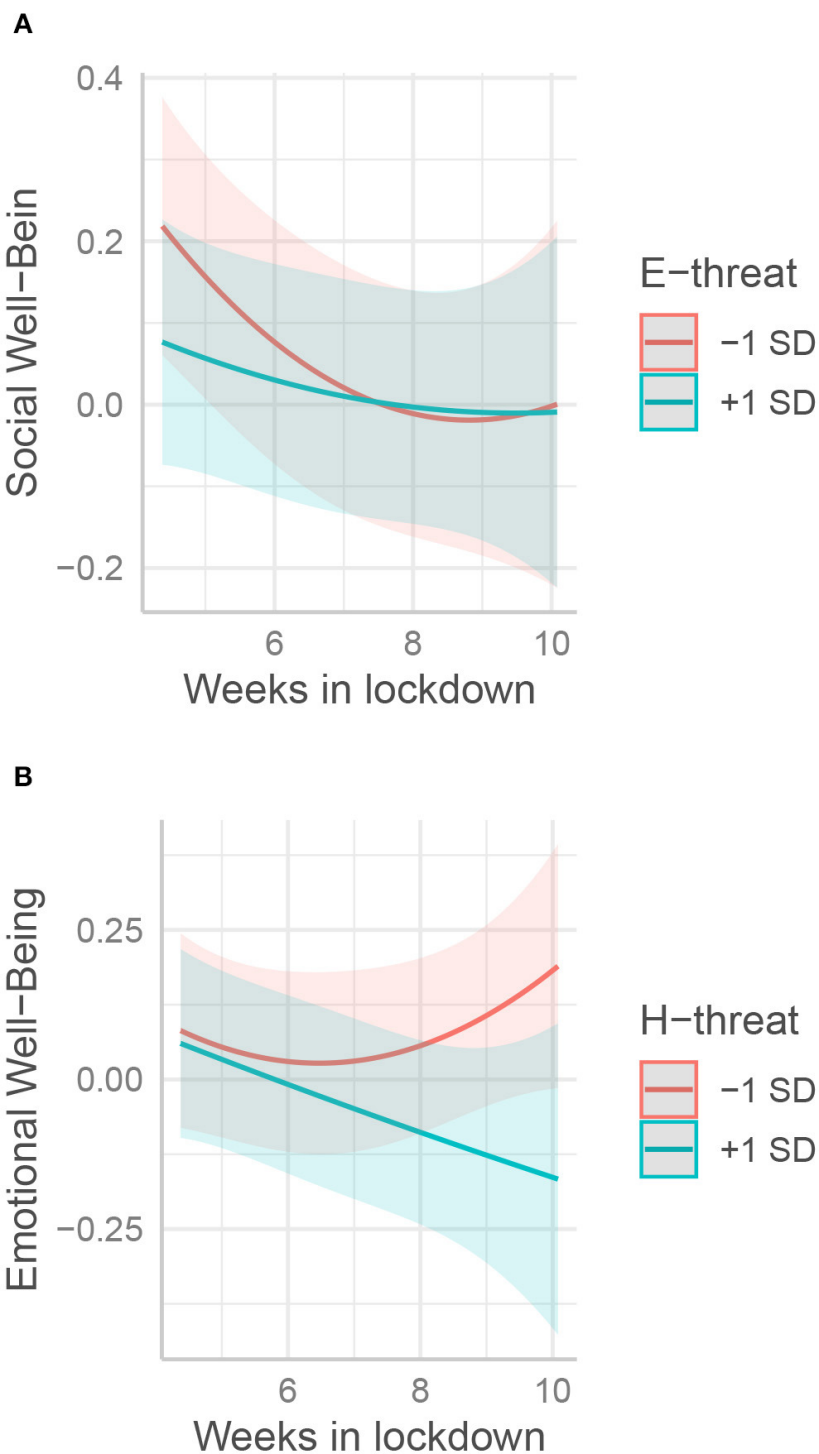
Interactions between the effects of reported economic **(A)** and health **(B)** threats and weeks spent in lockdown on well-being.

Although not predicted in our hypotheses, we tested whether the impacts of threats were more salient at a particular moment during lockdown. Economic threats reported as strong negatively impacted SWB at the outset. This effect diminished with time spent in lockdown so that, eventually, no differences were observed between individuals reporting high or low economic threats (Table 5, Step 3; Figure 3A). In other words, the negative impact on SWB of an economic threat was salient at the beginning, but not in the middle and at the end of lockdown. Conversely, the reported health threat had no particular impact on well-being at the outset, whereas with time spent in lockdown, the well-being of individuals who reported stronger threats decreased drastically in comparison with their peers (this pattern happened with EWB, SWB, and IWB, see Figure 3B for an illustration with EWB).

### Protective Effects of Psychological Resources on Well-Being

The following sections present the various results about how psychological resources protected well-being in the sample.

#### Psychological Resources Directly Affected Well-Being (H3)

The Step 2 columns in Tables 3–6 present the main effects of psychological resources on the various well-being variables. In agreement with H3, most psychological resources significantly and positively predicted well-being. Only self-transcendent wisdom and peaceful disengagement had no significant effect on any of the WB variables.

Also, as expected, the importance of a particular resource differed according to the well-being variable under consideration (H4). EWB was significantly predicted by hope, gratitude of being, and acceptance. PWB was significantly predicted by self-efficacy, personal wisdom, and gratitude of being. SWB was only significantly predicted by gratitude toward the world. Finally, IWB was significantly predicted by optimism and acceptance.

#### The Effect of the Time Spent in Lockdown on Well-Being Was Moderated by Psychological Resources (H4)

In Step 3, two-way interactions between time, reported threats, and psychological resources were additionally estimated. For better clarity, Tables 3–6 only report significant interactions.

**Figure 4:**
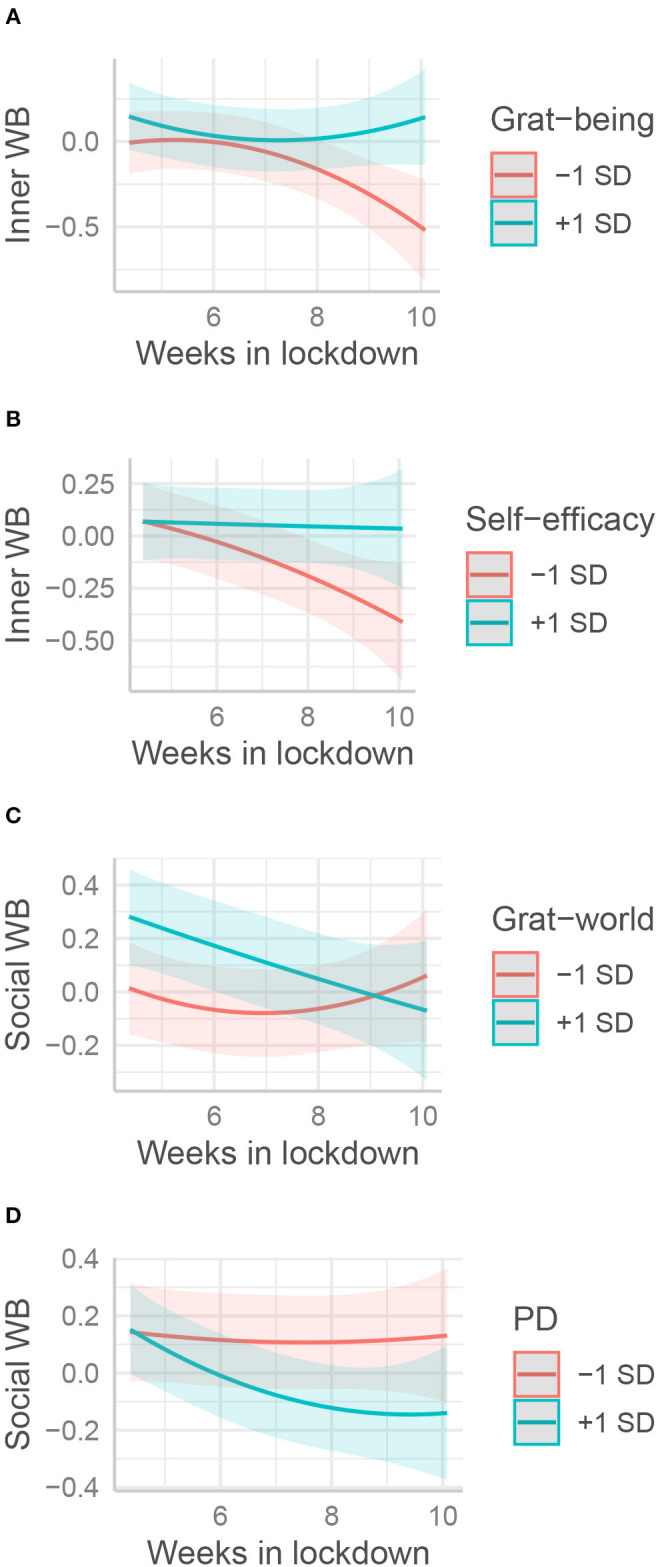
Interactions between the effects of psychological and weeks spent in lockdown on well-being. The moderation effects of **(A)** gratitude of being (Grat-being), **(B)** self-efficacy, **(C)** gratitude toward the world (Grat-world), and **(D)** peaceful disengagement (PD) on various form of well-being (WB) are displayed respectively. Psychological resources and well-being variables are standardized.

Gratitude for being alive was protective in reducing the negative time trend for PWB, SWB, and IWB, as was self-efficacy for IWB (Figures 4A,B). Reversely, the initial benefits provided by the disposition to feel gratitude toward the world for SWB vanished with time spent in lockdown (Table 3, see Figure 4C). Finally, and contrary to our hypothesis, peaceful disengagement appeared detrimental to all well-being variables: unlike their peers, participants who were more disengaged exhibited an overall decrease in other well-being variables as the lockdown proceeded (see Figure 4D for an illustration with SWB).

#### The Effects of Threats on Well-Being Were Moderated by Psychological Resources (H5)

**Figure 5:**
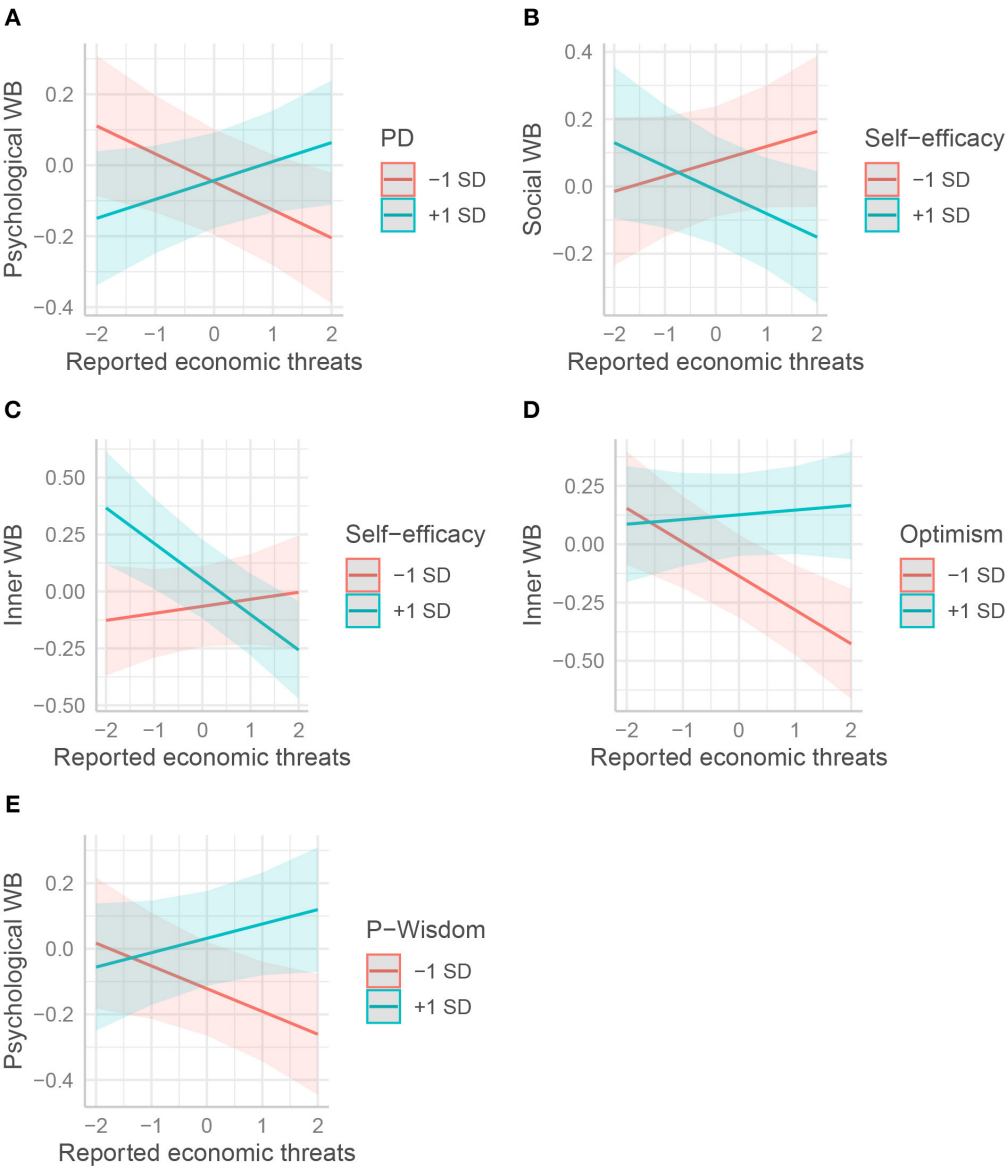
Interactions between the effects of psychological and reported economic threats on well-being. The moderation effects of **(A)** peaceful disengagement (PD), **(B,C)** self-efficacy, **(D)** optimism, and **(E)** personal wisdom (P-Wisdom) on various form of well-being (WB) are displayed respectively. All variables are standardized.

Results confirmed that the impact of threats was buffered by some of the psychological resources. Namely, four psychological variables reduced the negative effect of economic threat (Step 3 columns of Tables 3–6, Figure 5). Unexpectedly, peaceful disengagement was *disadvantageous* for PWB and SWB when the reported economic threat was low. It tended to be advantageous for PWB when it was high. Contrary to our hypothesis, for people with high self-efficacy SWB appeared to be negatively affected by economic threat, while people with low self-efficacy were positively affected by it. In contrast, self-efficacy was advantageous for IWB only when the reported economic threat was low. On the other hand, optimism clearly had a protective effect on IWB: very optimistic people were not affected by economic threat, while less optimistic people were strongly and negatively affected by it. Personal wisdom protected PWB from economic threat in the same way as optimism. However, wise individuals were positively affected by the economic threat.

**Figure 6:**
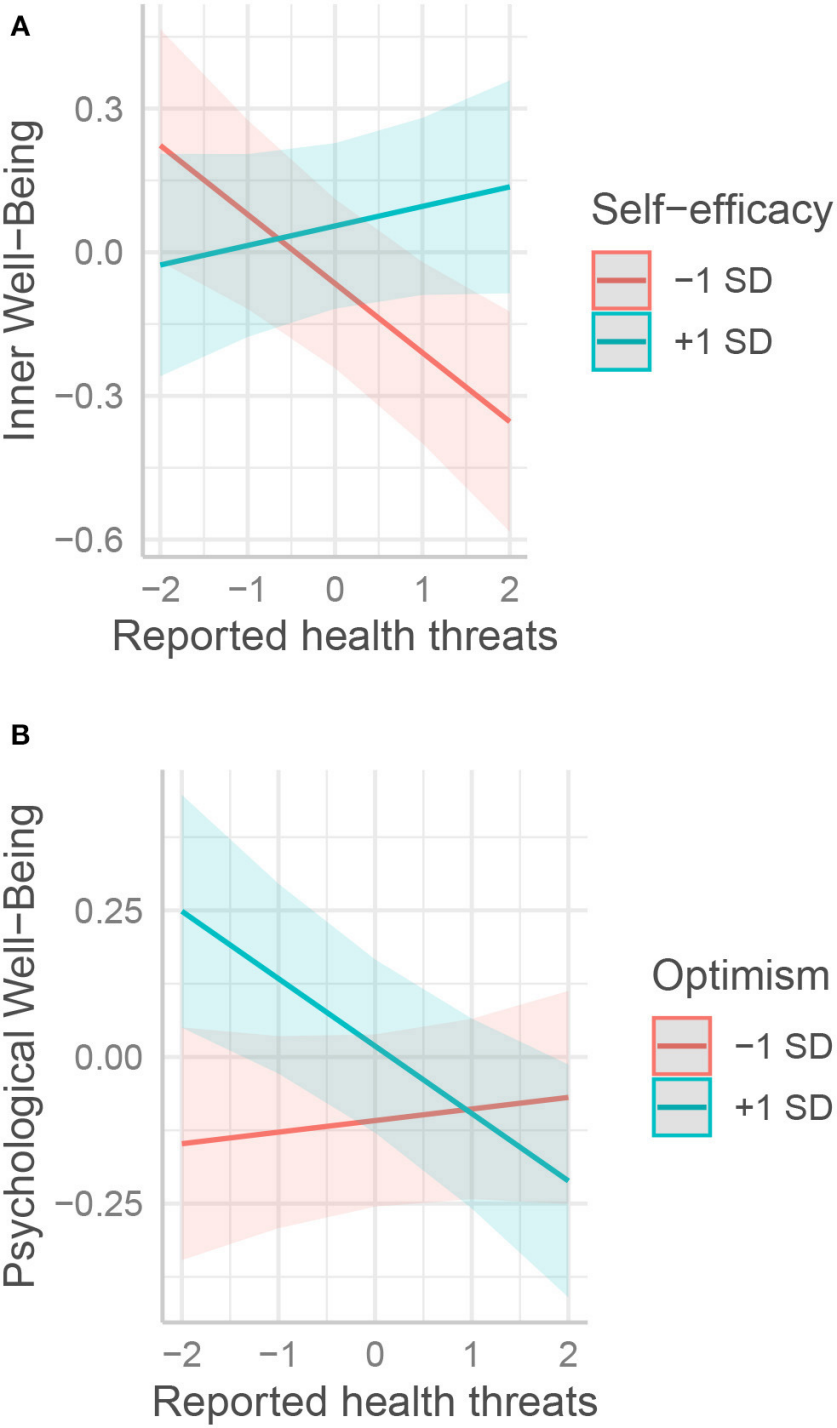
Interactions between the effects of psychological and reported health threats on well-being. The moderation effects of **(A)** self-efficacy, **(B)** optimism on various form of well-being are displayed respectively. All variables are standardized.

Two psychological resources interacted with reported threat to personal health and to relatives (Step 3 columns of Tables 3–6, Figure 6). Self-efficacy protected well-being (EWB, PWB, and IWB) against reported health threat: unlike their peers, those who scored high on this resource were not negatively affected by this threat (Figure 6A). On the contrary, the benefits of optimism on PWB disappeared when reported health threat became too high (Figure 6B).

#### Psychological Resources Directly Reduced Reported Threats and Indirectly Affected Well-Being Through the Decrease in Reported Threats (H6)

**Figure 7:**
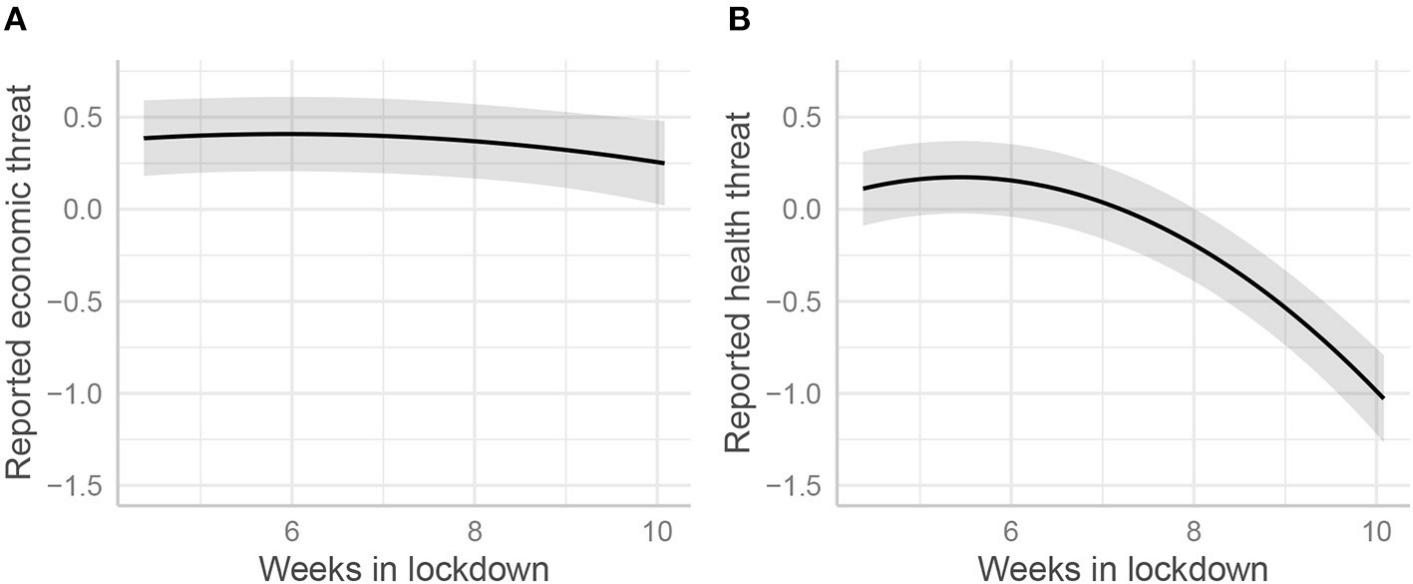
Standardized scores of reported economic **(A)** and health **(B)** threats as a function of the time spent in lockdown.

The effects on reported threat of time under lockdown, and of psychological resources are displayed in Table 7. Interestingly, economic threat was not affected by the time spent in lockdown, but health threat was: the reported health threat decreased strongly over time (see Figure 7). Hope was the only resource to downsize the reported economic threat. Unexpectedly, self-efficacy reinforced it. As for the threat to health, reported economic threat was negatively associated with optimism and positively with women.

**Table 7:** Standardized estimates of the effects of time in lockdown and psychological resources on reported health and economic threat.

	**Economic threat**			**Health threat**		
	***b* [95% CIs]**	**VIF**	**Zero-order**	***b* [95% CIs]**	**VIF**	**Zero-order**
Intercept	0.27 [−0.15; 0.69]			−0.58^**^ [−0.98; −0.17]		
Time	−0.81 [−1.76; 0.13]	1.00		−8.41^***^ [−9.52; −7.29]	1.00	
Time^2^	−0.79 [−1.72; 0.15]	1.00		−4.74^***^ [−5.84; −3.64]	1.00	
Age	0.02 [−0.07; 0.11]	1.26		−0.05 [−0.14; 0.04]	1.26	
Gender-women	0.06 [−0.14; 0.27]	1.04		0.31^**^ [0.10; 0.51]	1.04	
Income-middle	−0.26^*^ [−0.51; −0.02]	1.14		0.00 [−0.24; 0.24]	1.14	
Income-high	−0.38^**^ [−0.64; −0.11]	2.11		0.12 [−0.13; 0.38]	2.11	
Income-very high	−0.63^***^ [−0.89; −0.37]	1.89		−0.02 [−0.27; 0.23]	1.90	
Optimism	0.05 [−0.07; 0.16]	2.17	−0.05	−0.13^*^ [−0.24; −0.02]	2.18	−0.14^***^
Self-efficacy	0.14^*^ [0.02; 0.25]	1.59	−0.01	0.09 [−0.02; 0.20]	1.59	−0.03
Hope	−0.22^***^ [−0.34; −0.11]	1.70	−0.15^***^	−0.02 [−0.14; 0.09]	1.70	−0.09^*^
ST-Wisdom	0.04 [−0.06; 0.14]	2.27	−0.07	−0.04 [−0.13; 0.06]	2.27	−0.04
P-Wisdom	−0.04 [−0.15; 0.06]	2.14	−0.01	0.05 [−0.05; 0.15]	2.14	−0.10^*^
Grat-world	0.00 [−0.12; 0.12]	1.52	−0.07	−0.02 [−0.14; 0.09]	1.52	−0.09^*^
Grat-being	−0.02 [−0.13; 0.10]	2.14	−0.07	0.06 [−0.06; 0.17]	2.14	−0.11^**^
PD	−0.07 [−0.17; 0.03]		−0.10^*^	−0.04 [−0.13; 0.06]		−0.10^*^
Acc	0.02 [−0.10; 0.14]		−0.05	−0.12^*^ [−0.23; 0.00]		−0.14^***^
Marginal *R*^2^	0.09			0.10		
Conditional *R*^2^	0.80			0.73		

The column VIF displays the variance inflation factor. The column “Zero-order” displays unstandardized betas of resource predictors and their interactions, when all other resources are not included in the model. Reference level for income estimates: very low income (<1,000 euros per month). ST-Wisdom, self-transcendent wisdom; P-Wisdom, personal wisdom; Grat-world, gratitude toward the world; Grat-being, gratitude of being; PD, peaceful disengagement; Acc, acceptance.

* p < 0.05;

** p < 0.01;

*** *p < 0.001*.

We then tested whether hope, optimism, and acceptance would have an indirect impact on well-being variables through lessening reported threats. Self-efficacy indirectly and negatively affected EWB (β = −0.01, Confidence Intervals (CIs) = [−0.02;.00], *p* < 0.05, %_*mediated*_ = 13.28), PWB (β = −0.01, CIs = [−0.02;0.00], *p* < 0.05, %_*mediated*_ = 12.77), SWB (β = −0.01, CIs = [−0.02;0.00], *p* < 0.05, %_*mediated*_ = 12.86), and IWB (β = −0.01, CIs = [−0.02;0.00], *p* < 0.05, %_*mediated*_ = 15.62) through reported health threats. Hope indirectly and positively affected EWB (β = 0.01, CIs = [0.003;0.3], *p* < 0.01, %_*mediated*_ = 8.45) and IWB (β = 0.02, CIs = [0.01;0.03], *p* < 0.01, %_*mediated*_ = 18.60) through reported economic threats. Optimism indirectly and positively affected PWB (β = 0.01, CIs = [0.00;0.01], *p* < 0.05, %_*mediated*_ = 10.50), IWB (β = 0.01, CIs = [0.00;0.02], *p* < 0.05, %_*mediated*_ = 6.95) through reported health threats. Note that, although these mediation effects were statistically significant, the effect sizes are very small (maximum β = 0.02).

## Discussion

This study was designed to evaluate the putative protective effects of psychological resources on adults' well-being during lockdown. We expected that the amount of time spent under lockdown would affect well-being (H1), and that this effect would be reinforced by reported threats (H2) to health or economic situation. More importantly with regard to the goal of the present study, our main prediction was that possessing psychological resources would have a range of positive protective effects against the psychological damage of lockdown and the associated reported threats (H3–H6).

Our results mostly confirmed our hypotheses. First, according to H1, the levels of most well-being variables decreased with time (negative linear trends) and only started to bounce back when the French authorities announced the forthcoming end of lockdown, producing U-shaped curves (positive curvilinear components). Inner Well-Being (IWB) was the only variable not to bounce back. For emotional well-being, the linear trend was not significant. Second, according to H2, economic and health threats degraded all well-being variables except EWB, for which health threat negative effects did not reach significance. Only the interaction between health threat and linear time was significant. This is probably because reported health threat significantly decreased with time during lockdown, as Figure 7 shows. It could be seen as a logical effect of the lockdown, the genuine role thereof being to protect health. With H1 and H2 satisfied, we therefore knew that lockdown and threats influenced the various forms of well-being. This allowed us to test our remaining hypotheses regarding the effects of various forms of psychological resources. Results confirmed that psychological resources were beneficial for well-being.

- They directly and positively influenced well-being averages (H3).
- They moderated the trend of the well-being curve in a protective fashion (H4),
- They directly moderated the negative impact of reported threats (H5)
- They reduced reported threats and thus positively affected well-being indirectly (H6).

However, the latter hypothesis was only supported by small, albeit statistically significant, indirect effects, so we will not comment further on this issue without immediate pragmatic utility.

### The Protective Roles of Psychological Resources

Interestingly, and as suspected, psychological resources affected the various well-being dimensions differently, which corroborates in a new way the multidimensional nature of WB (e.g., Gallagher et al., 2009). EWB was positively predicted by hope, gratitude of being, and, to a lesser extent, by acceptance, PWB by self-efficacy, personal wisdom, and gratitude of being, SWB only by gratitude toward the world, and IWB by optimism, gratitude of being, and acceptance.

We now focus on the effects found for each psychological resource. Before commenting on each of the effects, we present a summary of the main effects found for each resource. Self-efficacy positively predicted PWB directly, reduced the negative impact of reported health threat on PWB and IWB, and was particularly beneficial to inner well-being when the reported economic threat was low. Compared to pessimists, optimists' IWB was generally higher and less influenced by reported economic threat. Hope positively predicted EWB and reduced reported economic threat. Personal wisdom was beneficial for PWB and moderated the effects of reported economic threats. Self-transcendent wisdom did not impact any WB variables when other resources were controlled. Gratitude toward the world was directly beneficial to social well-being. Gratitude of being positively and directly influenced EWB and PWB. Peaceful disengagement was not directly related to a WB variable, was beneficial only when the reported economic threat was high but was detrimental when the threat was low and also negatively influenced all slopes of WB over time. Finally, acceptance positively directly influenced EWB and IWB. We now discuss the effect of each of the psychological resources.

Self-efficacy was beneficial for well-being in multiple ways. First, high self-efficacy seemed to directly and positively influence psychological well-being during the lockdown. This is not entirely surprising, as psychological well-being includes an aspect of environmental mastery that is closely related to general self-efficacy (Ryff and Keyes, 1995). Nevertheless, only one item specifically addresses this dimension in the Mental Health Continuum questionnaire. Second, high self-efficacy protected PWB and IWB from the negative influence of feeling a health threat (Figure 6A). This might indicate that people with high self-efficacy felt that they could cope with this threat, maybe by engaging in adequate protective behaviors such as wearing masks and taking preventive measures. Coping with this threat may protect inner well-being (for example, by diminishing threat-related anxiety) and the sense of mastering one's environment. Third, interestingly, the conjunction of a low level of reported economic threat and a high feeling of self-efficacy seems to produce higher levels of inner well-being (Figure 5C). Further investigation would be needed on this because it might be that Inner Peace based on the simple lack of trouble, which could be grounded on the philosophical tradition of *ataraxia* and *apatheia*, resists health and economic threats differently from Inner peace based on inner control practices such as meditation. For example, Fredrickson et al. (2008) showed that loving-kindness meditation could enhance psychological resources. Dambrun et al. (2019) found that body-scan meditation can enhance happiness as measured by the SA-DHS scale from which the five items of our Inner Well-Being were taken. These research studies show that psychological resources can be developed to enhance well-being and Inner well-being in particular.

Optimism was directly beneficial to IWB but to no other well-being variable. This might be explained by the fact that high-optimism people experience fewer negative emotions (Carver et al., 2010) here in relation to the issue of the pandemic situation. In turn, this may lead to greater inner peace. Noteworthy, the MHC-SF is oriented toward positive mental health. Using another tool to measure negative aspects of emotional well-being might have placed more emphasis on negative emotions (e.g., Diener et al., 2009). Optimists' inner peace was not affected by economic threat, whatever its level (Figure 5D), possibly because they had a higher expectation that economic problems would be resolved one way or another.

Higher levels of hope were associated with higher levels of EWB. The most intuitive explanation is that, despite being in lockdown, high hope people find new ways to attain their different goals, and thus to be more satisfied with their present situation.

Personal wisdom was only significantly associated with psychological well-being (but marginally with SWB). It also appeared that personal wisdom acted as a protective variable against economic threat for PWB (Figure 5E). This result is not surprising, given prior empirical evidence about their relationships (Zacher and Staudinger, 2018). However, the lack of relation with EWB is not in line with those research findings. Again, it might be that threats influence emotional well-being through negative affects only, as it has been demonstrated that positive and negative affect are distinct dimensions with different correlates (Diener and Emmons, 1984; Raufaste and Vautier, 2008; Işik and Üzbe, 2015). The construct of emotional well-being would have been captured more comprehensively if we had incorporated such a dimension. Ardelt (2019) hypothesized that wisdom effects on well-being would be stronger during times of hardship by improving acceptance and gratitude. Controlling for both measures of gratitude and acceptance may attenuate the relationship between wisdom and satisfaction. The analysis of alternative models with our data provided useful insights. Personal wisdom was significantly related to emotional and social well-being only when all other personal resources were not controlled for (with the notable exception of self-transcendence and peaceful disengagement, though). This may indicate that personal wisdom can act as a “meta-resource,” promoting the development of other resources that, in turn, can enhance well-being. Because wise individuals seek to understand how to live a good life for themselves and for others (i.e., the cognitive and affective dimensions), they will tend to ameliorate their own behavior and cognition in order to grow (i.e., the reflective dimension). For example, by assessing one's own experience and/or referring to scientific or philosophical work, a person may come to see a particular worldview as beneficial to themselves and others. This person then seeks to adopt and cultivate the cognitive habit of interpreting situations and acting according to that worldview. In our data, interestingly, personal wisdom was not related to inner peace, whether or not self-transcendence was controlled for. All these findings will have to be confirmed by further studies, hopefully in other contexts.

Apparently, self-transcendent wisdom directly influenced none of the well-being variables. As for personal wisdom, this contradicts previous research findings (Aldwin et al., 2019). We performed the same analysis as with personal wisdom to explore whether self-transcendence effects were mediated by the other resources. Self-transcendent wisdom was significantly related to EWB and SWB when all other resource variables were not controlled for. Its relations with PWB and IWB were significant when controlled for personal wisdom only. This may also indicate that self-transcendent wisdom might also act as a meta-resource, which allows for the development of others. As self-transcendent wisdom is somewhat remote from the mundane, conventional view of things in Western countries, this lack of direct effects on well-being might be interpreted as a floor effect: the average participant may simply not have accumulated enough transcendent wisdom to make its direct effects detectable. Another possibility might be that this form of wisdom is more a form of “end-result” than something capable of influencing other variables.

Gratitude toward the world was the only resource to be significantly related to SWB. This indicates that this type of gratitude is particularly important for individuals to feel involved and cared for by people and society at large. It did not significantly predict any other well-being variable, however. This contradicts previous studies that highlighted relationships with both hedonic and eudaimonic well-being (Wood et al., 2010). It may be that the other gratitude variable, which shares a similar attitude toward an appreciation of life, captured these relationships instead. Indeed, when gratitude of being is not included in the models, gratitude toward the world effects become significant, for both emotional and psychological well-being. The effect on SWB appeared to be conditioned by the time spent in lockdown. Highly grateful individuals initially experienced a higher SWB, but this effect was attenuated during the lockdown, so that, at the end thereof, there was no difference with less grateful individuals. One of the conditions for the emergence of gratitude is the variety of life circumstances in which it can be experienced (McCullough et al., 2002). Thus, it may be that the decrease in social interactions due to the lockdown reduced the possibility for grateful people to experience gratitude, and thus to enjoy its benefits.

Gratitude for the simple fact of being seemed to be one of the best predictors of well-being. It was directly associated with EWB and PWB (and marginally significantly with SWB and IWB). In particular, the relationship with EWB was the strongest effect between one psychological resource and well-being in this study (β = 0.20). It also appeared that gratitude for being protected SWB and IWB mostly at the beginning of lockdown (Figure 4C). This confirms what we suspected, that a minimalist style (Kan et al., 2009) would be particularly relevant in a lockdown situation, when normal and social activities are drastically decreased. Contrary to the previous type of gratitude, people can rely on this resource at any time because it does not depend on external circumstances such as receiving social support. However, this may apply to the dimension of gratitude of a minimalist style, not to peaceful disengagement.

Peaceful disengagement did not directly predict any of the resources. Worse, it appeared that people who were more peacefully disengaged saw their well-being decrease more over time (Figure 4D). This applies to all well-being variables. In addition, when interacting with economic threat, it appeared to be beneficial to people reporting high economic threat, but, conversely, to be detrimental to people reporting low economic threat (Figure 5A). These results suggest that peaceful disengagement might be seen as an avoidance of a personal goal, promoting the use of avoidance coping strategies and thus reducing well-being (Elliot et al., 2011).

Finally, acceptance was positively associated with EWB and IWB. As for gratitude of being, these effects did not interact with time in lockdown. This result also confirmed the importance of this disposition in extreme situations (e.g., Nipp et al., 2016). Acceptance appears to be a powerful strategy that has the particular advantage of being beneficial regardless of external circumstances.

### Limitations and Future Directions

This study has several limitations. First, the use of self-reported questionnaires may have reduced the validity of the results. Participants' responses may have been altered by social desirability bias (Krumpal, 2011) or retrospective bias (Stone and Shiffman, 2002). Second, because our sample only comprised French residents, cultural differences might have affected the results. We hope that comparable studies will be published from other countries under lockdown. Third, the study accounted for a panel of nine resources. However, some other important psychological resources that were not accounted for in this study may have been important predictors of well-being. To name a few, mindfulness (Baer et al., 2004), equanimity (Juneau et al., 2020), and, more generally, all the character strengths and virtues widely studied in positive psychology after the seminal work by Peterson and Seligman (2004) would have been interesting to evaluate as protective factors. Fourth, despite some qualities of the longitudinal study in terms of power (470 individuals, six waves of measurement) and timing (assessment of resources and baseline well-being at the beginning of lockdown, then follow-up until the end of lockdown), we cannot ascertain causality. Although the lockdown situation might be construed as some sort of manipulation of people's freedom to move from their homes, this by no means constitutes an experiment: it was not possible to set up a control group or to randomize participants across the groups. At a deeper level, we saw that reported health threats decreased during the time course of the lockdown. Although we have no data to support this speculation, the lockdown and pandemic situation might also have affected the resources themselves. A dramatic—and relevant—example of this is provided by the online study of changes in character strengths after the 9/11 terrorist attacks on New York and Washington (Peterson and Seligman, 2003); the authors observed changes in some character traits related to the present study, namely, hope and gratitude. That said, the very fact that those resources may change suggests that we could take advantage of quiet times to prepare ourselves, to educate our minds, to accumulate a capital of psychological resources than could be tapped when hard times come. A promising avenue of research will be to test the dynamic relationships between different resources in long periods of time. In particular, as others have hypothesized (e.g., Ardelt, 2019), wisdom may act as a meta-motivational resource that serves to promote other resources for one's own and others' well-being.

## Conclusion

The lockdown situation experienced by half of the world population in the spring of 2020 was unprecedented. Leaving aside the inevitable grief induced in the victims' relatives, or in severely affected patients who eventually recovered, psychological damage may extend to all people forced to stay home—sometimes in highly uncomfortable situations—or to economically disadvantaged persons. This study sought to provide data to enhance the development of psychological resources in normal times to serve as a protection of individuals' well-being in times of crisis. What psychological assets should training target if one is to prepare for future pandemics? In this longitudinal study, we followed 470 confined French citizens for 8 weeks, until the end of the French lockdown. Results suggest that if emotional well-being were targeted, one would prepare by reinforcing hope and gratitude of being alive. If psychological well-being is targeted, one might work on self-efficacy, personal wisdom, and gratitude for being alive. For social well-being, a key could be gratitude toward the world. Finally, if inner well-being (peace of mind) is sought, working on optimism and acceptance could be the way.

## Data Availability Statement

The datasets presented in this study can be found in online repositories. The names of the repository/repositories and accession number(s) can be found : https://osf.io/45aq3.

## Ethics Statement

The studies involving human participants were reviewed and approved by Comité d'Ethique de la Recherche (CER)—Université Federale de Toulouse. The patients/participants provided their written informed consent to participate in this study.

## Author Contributions

NP and ER designed the study and materials, interpreted the results, wrote the manuscript, and both accept its final version. NP collected the data and analyzed the results. All authors contributed to the article and approved the submitted version.

## Conflict of Interest

The authors declare that the research was conducted in the absence of any commercial or financial relationships that could be construed as a potential conflict of interest.

## Footnotes

^1^To investigate the potential causes of “missingness” in the longitudinal design, we tallied the number of waves not responded to by each participant, from zero to five. Of all the study variables, including demographics, baseline levels of well-being, and psychological resources, only Age and Gender significantly predicted missingness: younger individuals were more likely to drop out (*b =* −0.02; *p* < 0.01), and men dropped out more often than women (*b =* −0.40; *p* < 0.05).
